# Thalamus Segmentation from Diffusion Tensor Magnetic Resonance Imaging

**DOI:** 10.1155/2007/90216

**Published:** 2007-10-29

**Authors:** Ye Duan, Xiaoling Li, Yongjian Xi

**Affiliations:** Department of Computer Science, College of Engineering, University of Missouri-Columbia, Columbia, MO 65211-2060, USA

## Abstract

We propose a semi-automatic thalamus and thalamus nuclei segmentation algorithm from Diffusion Tensor Magnetic Resonance Imaging (DT-MRI) based on the mean-shift algorithm. Comparing with existing thalamus segmentation algorithms which are mainly based on K-means algorithm, our mean-shift based algorithm is more flexible and adaptive. It does not assume a Gaussian distribution or a fixed number of clusters. Furthermore, the single parameter in the mean-shift based algorithm supports hierarchical clustering naturally.

## 1. INTRODUCTION

Thalamus is the relay center for nerve impulses in the brain. It mediates communication among sensory, motor, and associative brain regions. Axons from almost every sensory system connect here as the last site before the information reaches the cerebral cortex. Information received from the diverse brain regions is passed on to the cortex through the thalamus. Anatomically, thalamus is the largest, most internal structures of the diencephalon consisting of dual lobe masses of gray matter. It is located at the rostral end of the mid brain on each side of the third ventricle. Each lobe is about 4 centimeters. Motor nuclei of the thalamus receive signals from the striatum, cerebellum, project into the motor, and premotor areas of the cerebral cortex. The thalamus play a major role in the regulation of consciousness, alertness, arousal, and attention and is thus considered part of the limbic system.

Thalamus and thalamus nuclei segmentation have become more and more essential for a wide range of clinical and research applications. For example, thalamus changes in terms of volume and intensity are involved in a large number of diseases, such as schizophrenia, Parkinson’s disease, and multiple sclerosis. Conventional imaging modalities such as computerized tomography (CT) or magnetic resonance imaging (MRI) however, do not provide the necessary image contrast to differentiate the individual thalamic nuclei. On the other hand, a new noninvasive imaging modality diffusion tensor magnetic resonance imaging (DT-MRI) can relate the image intensities to the relative mobility of tissue water molecules [1]. In DT-MRI, a tensor describing local water diffusion is calculated for each voxel from measurements of diffusion in several directions. Since water diffusion along neural fiber tracts of the brain is highly anisotropic, DT-MRI had been used to study the brain connectivity by extracting the fiber tracts from the brain white matter. Most recently, researchers have started to use DT-MRI for segmentation purposes. Wiegell et al. [2] among of the first to segment thalamic nuclei directly from the DT-MRI data by using a k-means algorithm. Behrens et al. [3] proposed an algorithm to identify the thalamic nuclei by mapping the connections between the thalamus and the cortex. Jonasson et al. [4] presented a method for segmenting the thalamus and its subnuclei by propagating a set of coupled level sets through a region-based force defined from the similarity measure between the most representative tensor of each level sets and its neighboring voxels.

In this paper, we propose a semi-automatic thalamus and thalamus nuclei segmentation algorithm from diffusion tensor magnetic resonance imaging (DT-MRI) based on the mean-shift algorithm [5]. Comparing with existing thalamus segmentation algorithms which are based on K-means algorithm [2] or use K-means as an initialization [4], our mean-shift-based algorithm is more flexible and adaptive. It does not assume a Gaussian distribution or a fixed number of clusters. Furthermore, the single parameter in the mean-shift-based algorithm supports hierarchical clustering naturally. We will briefly review the background on diffusion tensor magnetic resonance imaging and mean shift clustering in Section 2. The main algorithm for thalamus and thalamus nuclei segmentation will be described in Section 3. Experimental results are shown in Section 4. Finally, the conclusion and some future work directions are discussed in Section 5.

## 2. BACKGROUND

### 2.1. Diffusion tensor imaging

Diffusion tensor magnetic resonance imaging (DT-MRI) is a recent MR imaging modality. In diffusion tensor MRI, a tensor describing local water diffusion is acquired for each voxel. The geometric nature of the diffusion tensors can quantitatively characterize the local structure in tissues such as bone, muscles, and white matter of the brain. A good review on DT-MRI can be found in [1, 6].

In general, the symmetric 3 by 3 diffusion tensor matrix D has six degrees of freedom (number of independent coefficients in a matrix representation). To estimate the tensor, then, at least six measurements (taken from different noncollinear gradient directions) are needed, in addition to the baseline image data $S_{0}$. Thus for each slice in the data set, seven images need to be collected with different diffusion weightings and gradient directions. Let $S_{0}$ represents the signal intensity in the absence of a diffusion-sensitizing field gradient and $S_{k}$ the signal intensity in the presence of gradient $g_{k}=\left(g_{k_{x}},g_{k_{y}},g_{k_{z}}\right),k=1,\ldots ,6.$ The equation for the loss in signal intensity due to diffusion is given by the Stejskal-Tanner formula:

(1) $$\text{ln}(S_{k})=\text{ln}(S_{0})-\gamma^{2}\delta^{2}(\Delta -\frac{\delta }{3})g_{k}^{T}Dg_{k},$$

where γ is the gyromagnetic ratio of hydrogen H (protons), δ is the duration of the diffusion sensitizing gradient pulses, and Δ is the time between the centers of the two pulses. The tensor D can then be computed by solving this system of six equations (1).

### 2.2. Mean shift clustering

Mean shift is a powerful general purpose technique for clustering scattered data [5]. Instead of assuming a fixed number of clusters as is common with other clustering methods (e.g. K-means), mean shift extracts the modes of the density function. We will review briefly the mean shift algorithm in the following. For a complete description of mean shift, please refer to the original paper [5].

Given an arbitrary set of n points $\chi =x_{1},\ldots ,x_{n}$ in the d-dimensional Euclidean space $R^{d}$. The multivariate kernel density estimate obtained with kernel K(x) and window radius h, computed in the point x, is defined as

(2) f^(x)=1nhd∑i=1nK(x−xih),

where K(x) is the spherically symmetric kernel function satisfying

(3) $$K(x)\geq 0,\;\int_{R^{d}}K(x)dx=1,$$

and h is a smoothing parameter called the bandwidth.

We can further define a profile function k(x) for the kernel function K(x) of (2) such that

(4) $$K(x)=c_{k,d}\,\;k(\|x{\|}^{2}),$$

where $c_{k,d}$ is the normalized constant. The density estimator of (2) can then be rewritten as

(5) f^(x)=ck,dnhd∑i=1n k(‖x−xih‖2).

Assume now that we are interested in subdividing scattered data χ into a set of clusters. It is natural to consider the points where f^ defined by (5) have local maxima as centers of the clusters. The simplest method to find the local maxima of the f^ is to compute the gradient of f^ and use a hill-climbing process to map each input point to its local maxima (i.e., mode) defined by f^. These resulting modes can then be used to select cluster shapes using basins of attraction, and can have very nontrivial shapes unlike k-means clustering where points are simply assigned to the nearest cluster center. The single bandwidth parameter h allows the number of clusters to be chosen in terms of a length scale in the input point space.

From (5) we can compute the gradient of f^:

(6) ∇f^(x)=2ck,dnhd+2∑i=1ng(‖x−xih‖2)m(x),

where $g(x)=-k^{\prime }(x)$. m(x) is the mean shift vector and is given by

(7) $$m(x)=\frac{\sum_{i=1}^{n}{\;x}_{i}g(\|\left(x-x_{i}\right)/h{\|}^{2})}{\sum_{i=1}^{n}\;g(\|\left(x-x_{i}\right)/h{\|}^{2})}-x,$$

for example, the difference between the weighted mean, using the kernel g for weights, and x, the center of the kernel (window). The general mean shift clustering procedure consists of the following two steps:

1. initialize: $y_{0}=x\,;$
2. update by hill climbing: $y_{j+1}=y_{j}+m(y_{j})$ until convergence.

## 3. THALAMUS AND THALAMUS NUCLEI SEGMENTATION BY MEAN SHIFT

In this section, we will describe our framework for thalamus and thalamus nuclei segmentation from DT-MRI data based on the previously described mean-shift algorithm. There are two different domains of similarity: spatial and tensor in the DT-MRI image data, for example, each point $x_{i}$ in the data set χ of the joint spatial-tensor domain has two components of different nature, $x_{i}=(x_{i}^{s},x_{i}^{r})$, where $x_{i}^{s}$ is the spatial component, and $x_{i}^{r}$ is the tensor component. The mean shift algorithm can be employed by extending the density estimator of (5) with the following separable kernels:

(8) f^(x)=C∑i=1nks(‖xs−xishs‖2)kr(‖xr−xirhr‖2),

here $k_{s}$ is the kernel profile in the spatial domain with bandwidth parameter $h_{s}$, $k_{r}$ is the kernel profile in the tensor domain with bandwidth parameter $h_{r}$, and C is the corresponding normalization constant. As suggested by [5], an Epanechnikov kernel with profile $k_{E}(x)$,

(9) $$k_{E}\left(x\right)=\{\begin{array}{ll} 1-x & \text{i}\text{f 0}\leq x\leq 1,\,\\ 0 & \text{otherwise}, \end{array}$$

or a (truncated) normal kernel with profile $k_{N}\left(x\right)=\text{exp}\left((-1/2)x\right),x>0,$ always provides satisfactory performance, so the user only has to set the bandwidth parameter $h=\left(h_{s},h_{r}\right)$, which, by controlling the size of the kernel, determines the resolution of the mode selection. For DT-MRI data, we have tried both kernels and found no significant difference. In our experiment we choose the Epanechnikov kernel because of its simplicity. The distance metric in the spatial domain is the Euclidian distance metric, the distance metric in the tensor domain is the following Frobenius norm:

(10) $$\|x_{1}^{r}-x_{2}^{r}\|=\sqrt{\text{T}\text{r}\text{a}\text{c}\text{e}((D_{x_{1}}-D_{x_{2}}){\left(D_{x_{1}}-D_{x_{2}}\right)}^{T})},$$

$D_{x_{1}},D_{x_{2}}$ are the tensor matrices at points $x_{1},\;x_{2},$ respectively.

## 4. EXPERIMENTAL RESULTS ON REAL AND SYNTHETIC DATA

### 4.1. Synthetic data

To validate our method, we have applied our method on some synthetic data. For comparison purpose, we use the same synthetic data used in [4]. A slice of the synthetic tensor field is shown in Figure 6. In Figure 7, the regions have been segmented on the synthetic field without any noise added. In Figure 8, noise is added into the same synthetic field at signal-to-noise-ratio (SNR) level 32. In both of these two examples, our algorithm obtained much better results than both the K-means algorithm and the method of [4].

### 4.2. Real data

**Figure 1:**
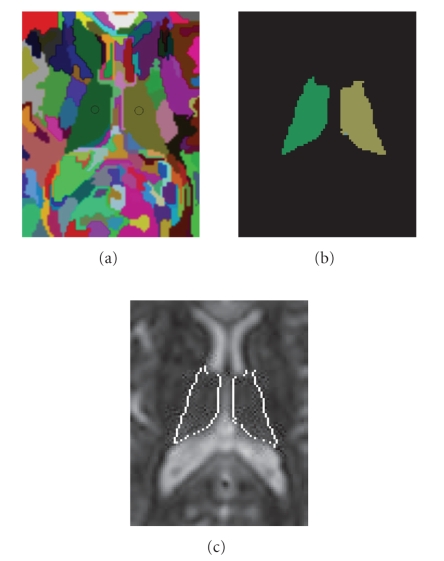
Thalamus segmentation. (a) The initial thalamus segmentation is conducted interactively, that is, the user need to identify the pair of symmetric clusters (left thalamus and right thalamus, marked by circles in the figure for illustration) from other clusters. Since the thalamus is bounded by relatively homogenous structures such as the fiber tract and Cerebrospinal fluid (CSF), this step can be done quite easily. (b) Extracted pair of thalamus. (c) Extracted pair of thalamus superimposed on the original DT-MRI image.

In this section, we will show some of the experimental results of thalamus and thalamus nuclei segmentation from DT-MRI data. Figure 1 illustrates the thalamus segmentation process. The initial thalamus segmentation is conducted interactively, for example, after applying the mean shift algorithm with a bigger bandwidth parameter, the user needs to identify the pair of symmetric clusters (left thalamus and right thalamus, marked by circles in Figure 1(a) for illustration) from other clusters. Since the thalamus is bounded by relatively homogenous structures such as the fiber tract and Cerebrospinal fluid (CSF), this step can be done quite easily (Figure 1(b)). In this example, the bandwidth parameter is set as $h=\left(h_{s},h_{r}\right)=(7,13).$

**Figure 2:**
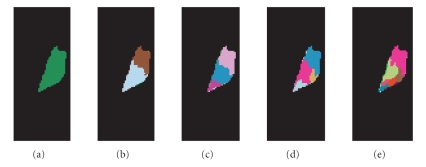
Mean shift based hierarchical thalamus nuclei segmentation results of the left thalamus. (a) The segmented left thalamus by setting the bandwidth parameter as $h=\left(h_{s},h_{r}\right)=\left(7,13\right).$ (b) The segmented anterior and posterior parts of the left thalamus by setting the bandwidth parameter as $h=\left(h_{s},h_{r}\right)=(7,11).$ (c) The segmentation result by setting the bandwidth parameter as $h=\left(h_{s},h_{r}\right)=(7,10.5).$ (d) The segmentation result by setting the bandwidth parameter as $h=\left(h_{s},h_{r}\right)=\left(7,10\right).$ (e) The segmented thalamus nuclei by setting the bandwidth parameter $h=\left(h_{s},h_{r}\right)=\left(7,8\right).$

The initially segmented thalamus will then serve as the mask for the subsequent thalamus nuclei segmentation, which will be conducted automatically with a smaller bandwidth parameter. The parameter chosen will determine the scale of features detected, so different values may be desired based on the data set quality, features of interest, and so forth. Figure 2 shows the thalamus nuclei segmentation results of the left thalamus. To illustrate the hierarchical nature of the mean-shift-based algorithm, we fixed the spatial bandwidth $h_{s}$ as 7, and gradually reduced the tensor bandwidth $h_{r}$ from 13 (Figure 2(a)) to 11 (Figure 2(b)), 10.5 (Figure 2(c)), 10 (Figure 2(d)), and 8 (Figure 2(e)). By gradually reducing the bandwidth, more and more detailed nuclei structures can be seen.

**Figure 3:**
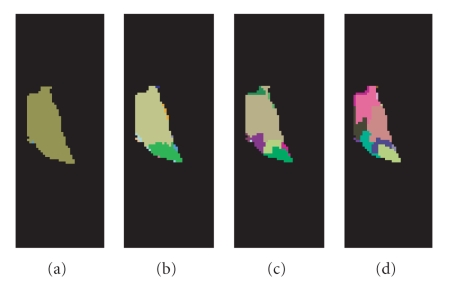
Hierarchical thalamus nuclei segmentation results of the right thalamus. (a) The right thalamus, $h=\left(h_{s},h_{r}\right)=\left(7,13\right).$ (b) The segmented anterior and posterior parts of the right thalamus, $h=\left(h_{s},h_{r}\right)=\left(7,11\right).$ (c) The segmentation result by setting the bandwidth parameter as $h=\left(h_{s},h_{r}\right)=\left(7,7\right).$ (d) The segmented thalamus nuclei by setting the bandwidth parameter $h=\left(h_{s},h_{r}\right)=\left(1,1.5\right).$

**Figure 4:**
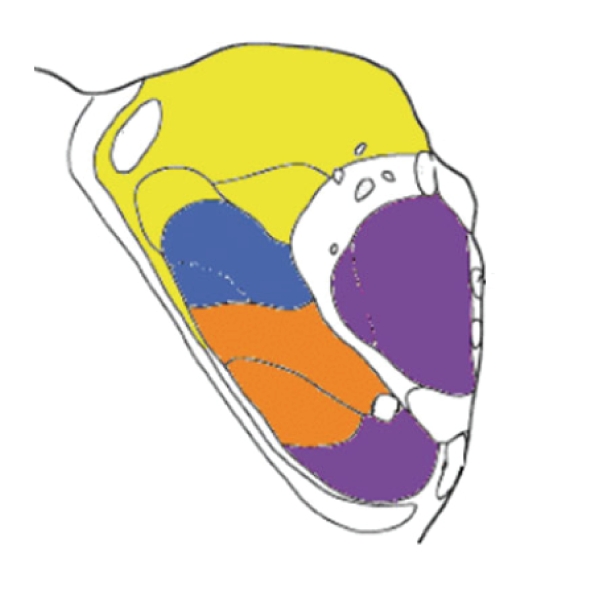
The histological atlas of the human thalamus with nuclei outlined by black lines (Image courtesy of Behrens et al. [3]).

**Figure 5:**
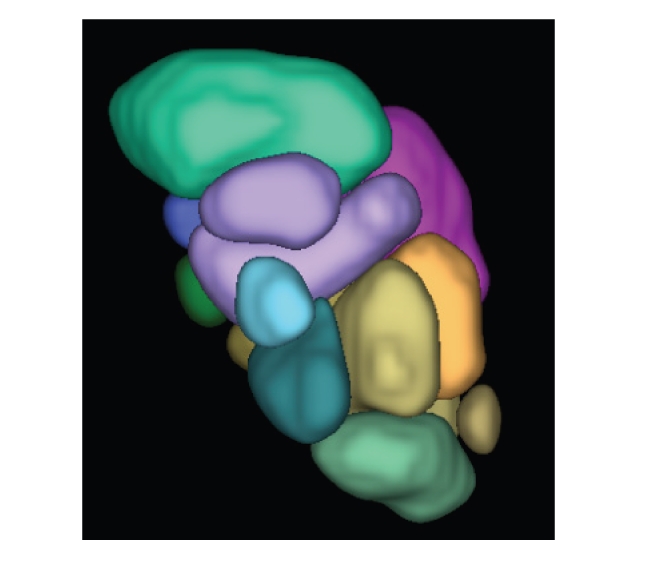
A 3D view of the thalamus nuclei segmentation results.

**Figure 6:**
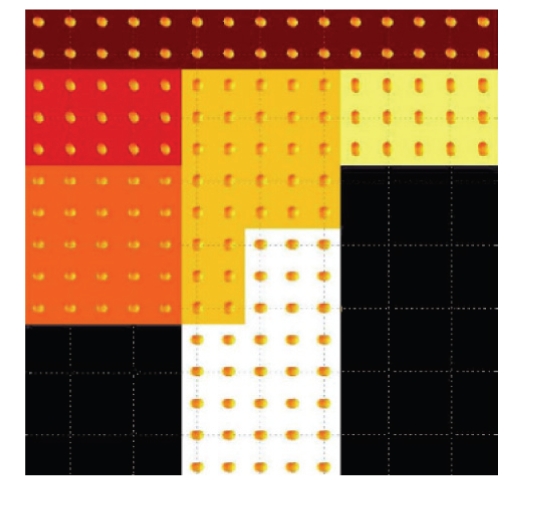
A slice of the synthetic tensor field used to test the segmentation method. (Image courtesy of Jonasson et al. [4].)

**Figure 7:**
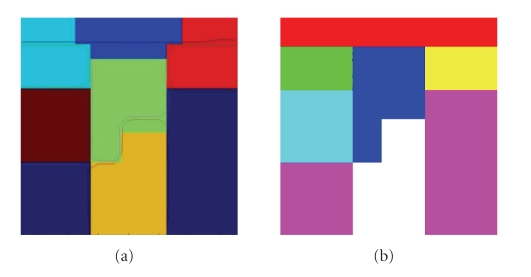
Segmentation results on a synthetic field, without any noise added. (a) Results of [4] displayed on the results obtained with the K-means algorithm. (b) Results from our algorithm.

**Figure 8:**
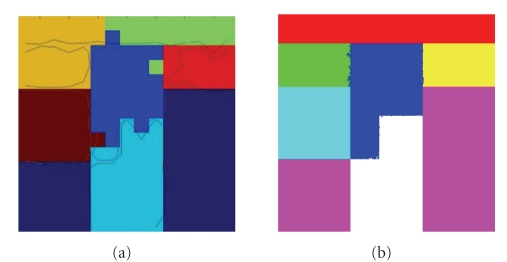
Segmentation results on a synthetic field, with SNR = 32. (a) Results of [4] displayed on the results obtained with the K-means algorithm. (b) Results from our algorithm.

We conducted similar hierarchical segmentation for the right thalamus as well and is shown in Figure 3. We again fixed the spatial bandwidth $h_{s}$ as 7, and gradually reduced the tensor bandwidth $h_{r}$ from 13 (Figure 3(a)) to 11 (Figure 3(b)), and 7 (Figure 3(c)). However, quite different settings of the bandwidth parameter need to be chosen $h=\left(h_{s},h_{r}\right)=\left(1,1.5\right)$ to obtain the desired final thalamus nuclei segmentation (Figure 3(d)). We do not know the exact reason of this (the different parameter setting for the left and right thalamus), one of the possible reasons might be the artifact during the image acquisition process (e.g., the image slice is not totally orthogonal across the thalamus), or the nuclei structures between the left and the right thalamus are not symmetrical indeed. More experiments and research are certainly needed in the future to answer these questions. Nonetheless, if we compare Figure 2(e) and Figure 3(d) with Figure 4, which is the histological atlas of the human thalamus with nuclei outlined by black lines [3], we can see they are very close. Finally, a 3D rendering of the thalamus nuclei segmentation result is shown in Figure 5.

## 5. DISCUSSION

The main contribution of the paper is the application of the powerful mean shift clustering algorithm for thalamus segmentation from the DT-MRI data. Comparing with existing thalamus segmentation algorithms ([2, 4]) which are based on K-means algorithm, our mean-shift-based algorithm has several potential advantages. (1) Since the mean shift algorithm is based on nonparametric density estimation, it does not assume the data is always Gaussian, hence it is more generic and flexible. (2) Unlike K-means algorithm, the mean-shift algorithm does not assume a fixed number of clusters, hence it is more adaptive to the diversity of the dataset. (3) There is only one parameter in the mean-shift-based algorithm, the bandwidth parameter, which controls the scale of the features detected. (4) And by setting the bandwidth parameter from large to small, mean shift naturally supports hierarchical clustering, as shown in this paper on thalamus and thalamus nuclei segmentation.

There are two main directions to further improve the thalamus segmentation results: (1) currently, the thalamus segmentation is conducted semi-automatically, that is, the user has to pick the pair of distinct clusters (left and right parts of the thalamus) from other neighboring clusters such as the fiber tracts and CSF. Although this is quite easy to do, it would be even better if the thalamus can be automatically segmented; moreover, a postprocessing active-contour-based diffusion step (as is done in [4]) might be able to further smooth the nuclei boundary obtained from the clustering algorithm. (2) We would like to work closely with domain specialists such as neurobiologists to verify and validate the segmentation results, and to identify the thalamus nuclei structures. The collaboration with domain specialists will also help us to choose the best bandwidth parameter for the mean shift algorithm to create clinically most meaningful segmentation results.
